# Noninvasive assessment of coronary vasodilation using cardiovascular magnetic resonance in patients at high risk for coronary artery disease

**DOI:** 10.1186/1532-429X-10-28

**Published:** 2008-05-30

**Authors:** Patricia K Nguyen, Craig Meyer, Jan Engvall, Phillip Yang, Michael V McConnell

**Affiliations:** 1 Department of Medicine, Division of Cardiovascular Medicine, Stanford University, Stanford, USA; 2 Department of Biomedical Engineering, University of Virginia, Virginia, USA; 3 Department of Clinical Physiology, Linköping Heart Center, Linköping, Sweden; 4 Department of Electrical Engineering, Stanford University, Stanford, USA

## Abstract

**Background:**

Impaired coronary vasodilation to both endothelial-dependent and endothelial-independent stimuli have been associated with atherosclerosis. Direct measurement of coronary vasodilation using x-ray angiography or intravascular ultrasound is invasive and, thus, not appropriate for asymptomatic patients or for serial follow-up. In this study, high-resolution coronary cardiovascular magnetic resonance (CMR) was used to investigate the vasodilatory response to nitroglycerine (NTG) of asymptomatic patients at high risk for CAD.

**Methods:**

A total of 46 asymptomatic subjects were studied: 13 high-risk patients [8 with diabetes mellitus (DM), 5 with end stage renal disease (ESRD)] and 33 age-matched controls. Long-axis and cross-sectional coronary artery images were acquired pre- and 5 minutes post-sublingual NTG using a sub-mm-resolution multi-slice spiral coronary CMR sequence. Coronary cross sectional area (CSA) was measured on pre- and post-NTG images and % coronary vasodilation was calculated.

**Results:**

Patients with DM and ESRD had impaired coronary vasodilation to NTG compared to age-matched controls (17.8 ± 7.3% vs. 25.6 ± 7.1%, p = 0.002). This remained significant for ESRD patients alone (14.8 ± 7.7% vs. 25.6 ± 7.1%; p = 0.003) and for DM patients alone (19.8 ± 6.3% vs. 25.6 ± 7.1%; p = 0.049), with a non-significant trend toward greater impairment in the ESRD vs. DM patients (14.8 ± 7.7% vs. 19.8 ± 6.3%; p = 0.23).

**Conclusion:**

Noninvasive coronary CMR demonstrates impairment of coronary vasodilation to NTG in high-risk patients with DM and ESRD. This may provide a functional indicator of subclinical atherosclerosis and warrants clinical follow up to determine prognostic significance.

## Background

Impaired vasodilation is an early marker of atherosclerosis [1-4]. Although abnormal response to endothelial-*dependent *stimuli is more commonly associated with coronary artery disease (CAD) [3,5-8], several studies have associated impaired vasodilatory response to nitroglycerin (NTG) with risk factors for coronary artery disease [9] and increased future clinical events [6,10]. Previous studies, however, have used x ray angiography [3-8] and intravascular ultrasound [11] which are invasive and, thus, not appropriate for asymptomatic patients or serial follow-up. A non-invasive measure of subclinical coronary atherosclerosis may help identify patients who are at increased risk and guide therapy toward reducing morbidity and mortality. We and others have previously developed a non-invasive method to measure NTG-induced coronary vasodilation with coronary cardiovascular magnetic resonance (CMR) [12,13]. We hypothesize that impaired coronary vasodilation to NTG can be demonstrated noninvasively in asymptomatic patients at increased risk for coronary artery disease.

## Methods

### Subjects

Asymptomatic patients with diabetes mellitus (DM, N = 8) and end stage renal disease (ESRD, N = 5), as well as age-matched controls (N = 33) were recruited consecutively. Subjects were excluded if they had a history of chest pain, coronary artery disease, myocardial infarction, stroke or peripheral vascular disease. Vasoactive medications were discontinued 24 hours before the examination. All subjects provided written informed consent approved by the Human Subjects Committee at Stanford University.

### CMR

A 1.5-T Signa MRI scanner (GE Healthcare, Milwaukee, Wisconsin) equipped with high-performance gradients (40 mT/m, 150 mt/m/ms) and a real-time interactive workstation were used. A commercial coil provided signal reception (5-inch General Purpose Coil, Model #2127316, GE Healthcare, Milwaukee, Wisconsin). Blood pressure and heart rate were monitored throughout the study (Omega 1400, In vivo Research, Inc., Orlando, Florida).

### Protocol

NTG-induced coronary vasodilation was performed as previously described [13]. A real-time interactive MR system [13-15] was used to localize coronary arteries (16 frames/sec, TR 4.6 ms, flip 30, slice thickness 7 mm, FOV 24 cm, in-plane resolution 2.7 mm). High-resolution coronary MRA was then performed using a cardiac-gated, breath-held, multi-slice spiral sequence [13] (FOV = 20–28 cm, slice thickness = 5 mm, TR = 1 heartbeat, TE = 7 ms, in-plane spatial resolution = 0.62–0.99 mm, 14 to 20 interleaves, flip angle = 60 degrees, acquisition gated to diastole). In-plane and cross-sectional images were acquired before and then 5 minutes after 0.4 mg sublingual NTG, which was given while the subject was in the magnet. Images were reconstructed onto a 512 × 512 matrix, yielding a pixel size of 0.39 to 0.55 mm. Real-time short axis views of the left ventricle (LV) from the apex to base as well as 4-, 3- and 2-chamber views were also obtained to evaluate LV function.

### Image Analysis

For quantitative analysis of coronary vasodilation, the cross-sectional right coronary artery (RCA) images were used, except in subjects with a small non-dominant RCA, where the cross-sectional left anterior descending artery (LAD) images (n = 5) were used. As described previously[13] the slice with the most circular cross-section was identified on the pre-NTG images and the corresponding post-NTG slice was carefully matched according to the surrounding cardiac and chest wall structures. These images were then pooled and randomized, with no patient information or NTG status provided on the images, and then analyzed independently and in a blinded fashion by one observer. A custom designed software program was used to analyze the cross sectional images: after images were magnified two-fold, an ovoid region of interest tool was used to trace around the RCA or LAD, yielding the cross-sectional area (CSA). This analysis has been shown previously to have a low intra- and inter-observer variability [12,13] and good correlation with x-ray coronary angiography [13].

### Statistical Analysis

Data were expressed as mean values ± standard deviation. The difference in coronary artery size before and after NTG was compared by a paired two-sided *t *test. Differences in % coronary vasodilation between patients and controls were tested using an unpaired two-sided *t *test. Differences among subject groups were tested using a one-way analysis of variance (ANOVA) and Fisher exact test. All statistical analyses were performed with StatView (version 5, SAS Institute Inc., Cary, NC).

## Results

Clinical characteristics of the patient and control groups are shown in Table 1. All 46 subjects completed the study without complications. NTG caused a small systemic effect: 9.1 ± 8.5% decrease in systolic blood pressure, 3.2 ± 6.7% decrease in diastolic blood pressure, and 5.4 ± 7.7% increase in heart rate, which was not different between patients and controls (Table 1). The mean age of the patients and controls was similar (patients: 55.3 ± 14.3 yrs, controls: 51.8 ± 10.4 yrs, p = 0.4); however, the sex difference was significant (patients: 92% male, controls: 48% male, p = 0.006). Patients with DM and ESRD had a higher incidence of hypertension and use of angiotensin converting enzyme inhibitors (p = 0.001). One control subject was excluded from analysis because of abnormal left ventricular function. No subjects needed to be excluded because of image quality.

**Table 1:** Baseline characteristics of study subjects

Characteristics	Controls (n = 33)	Patients with DM or ESRD (n = 13)	P value
Age (years)	51.8 ± 10.4	55.3 ± 14.3	0.4
Male	16/33	12/13	0.006
HTN	9/33	9/13	0.02
FH	6/33	5/13	0.15
Dyslipidemia	4/33	4/13	0.13
ACE-I	2/33	6/13	0.001
Current smoker	1/33	1/13	0.49
Decrease in SBP (mmHg)	9.2 ± 7.0%	9.0 ± 11.5%	0.97
Decrease in DBP (mmHg)	3.7 ± 7.0%	2.0 ± 5.5%	0.45
Increase in HR (beats/min)	5.5 ± 7.9%	5.2 ± 7.2%	0.89

Results are given as mean ± standard deviation or proportions. HTN: hypertension; FH: family history; ACE-I: angiotensin converting enzyme inhibitor; SBP: systolic blood pressure; DBP: diastolic blood pressure; HR: heart rate

**Figure 1:**
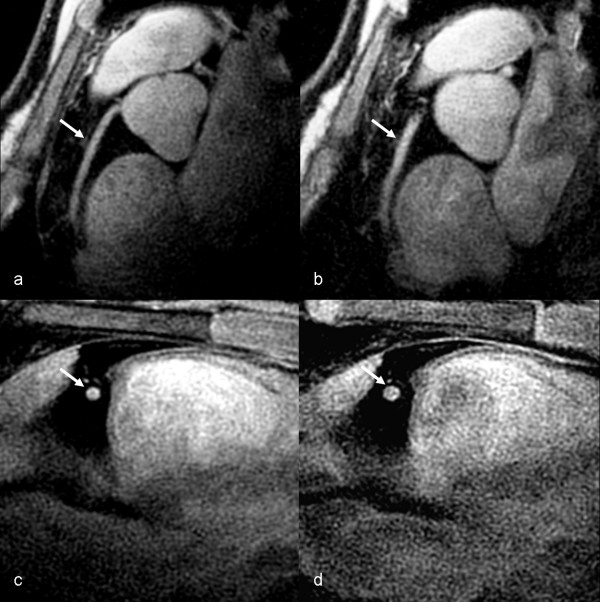
Coronary CMR of a control subject showing coronary vasodilation: In plane pre-NTG (a) and post-NTG (b) and cross-sectional pre-NTG (c) and post-NTG (d) images of the proximal right coronary artery (arrows).

**Figure 2:**
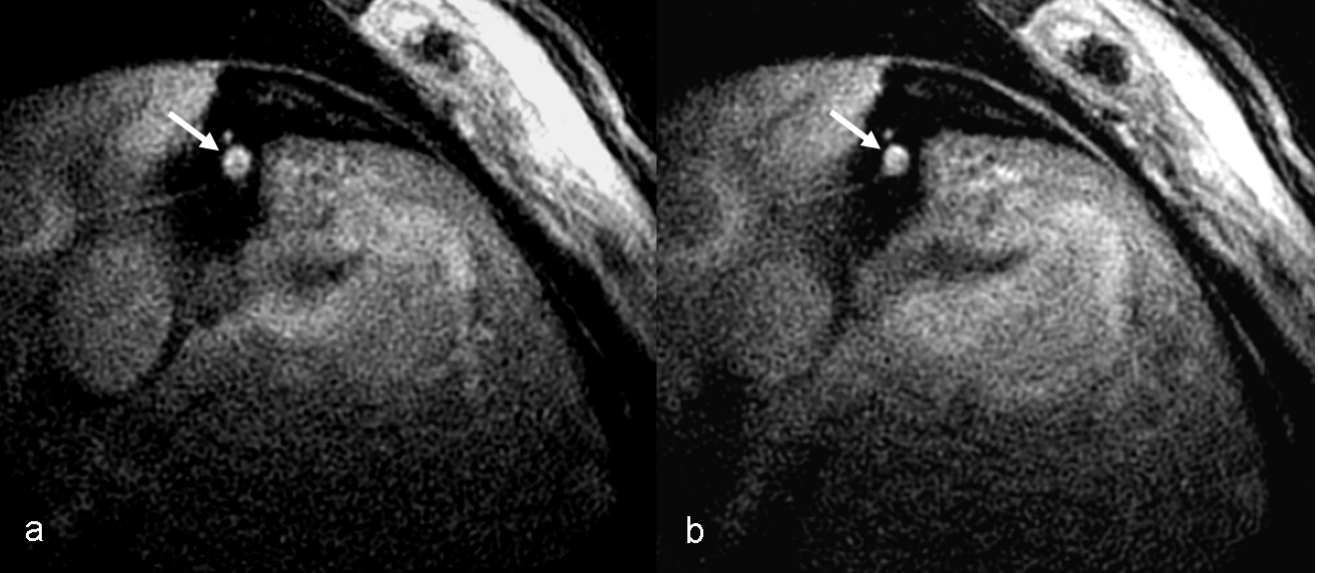
Coronary CMR of a patient with end stage renal disease showing no significant coronary vasodilation: Cross-sectional pre-NTG (a) and post-NTG (b) images of the proximal right coronary artery.

**Figure 3:**
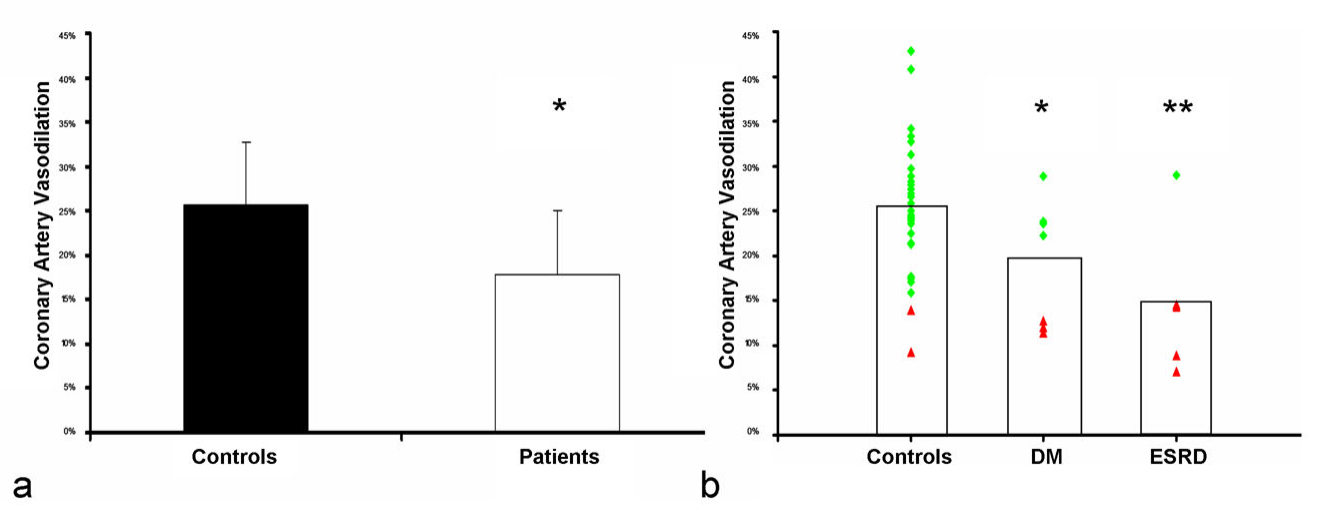
Comparison of the percent coronary vasodilation in all subjects: a) Compared to controls, patients had significantly impaired vasodilation. b) Patient subgroup analysis: Endstage renal disease (ESRD) patients and diabetes (DM) patients both had significantly impaired vasodilation compared to controls. Individual subjects with vasodilation < 15% indicated in red. * – p < 0.05 vs. controls, ** – p < 0.005 vs. controls.

Coronary CMR detected a significant increase in coronary CSA with NTG in both patients and controls (p < 0.0001 for both), but the degree of coronary artery vasodilation to NTG was significant impaired in patients compared to controls (17.8 ± 7.3% vs. 25.6 ± 7.1%, p = 0.002, Figures 1, 2, 3). This % vasodilation in controls was similar to that of our prior study (24.0%) [13]. Analysis by patient subgroup was also significant (ANOVA p = 0.005, Figure 3b), with ESRD patient alone vs. controls (14.8 ± 7.7% vs. 25.6 ± 7.1%; Fisher exact test p = 0.003) and DM patients alone vs. controls (19.8 ± 6.3% vs. 25.6 ± 7.1%; Fisher exact test p = 0.049) showing impaired coronary vasodilation. There was a non-significant trend toward greater impairment in the ESRD vs. DM patients (14.8 ± 7.7% vs. 19.8 ± 6.3%; Fisher exact test p = 0.23).

Given the disparity in gender distribution between the patients and controls, we analyzed by gender. The impairment of coronary vasodilation remained significant in male patients vs. male controls (18.3 ± 7.4% vs. 27.6 ± 5.7 %, p = 0.002). The lack of females in the patient group precluded comparing female patients to controls.

Comparing RCA and LAD, there were not significant differences in baseline CSA (RCA: 16.3 ± 4.2 mm^2 ^vs. LAD: 14.7 ± 2.6 mm^2^, p = 0.31) or coronary vasodilation (RCA: 23.5 ± 7.9% vs. LAD: 22.2 ± 8.2%, p = 0.77).

## Discussion

In the present study, noninvasive coronary CMRA demonstrated impaired coronary artery vasodilation to NTG in a group of patients at increased risk for coronary artery disease. To our knowledge, this is the first study to use *noninvasive imaging *to directly assess impaired *epicardial coronary vasodilation *in this patient group.

### Coronary Vasomotor Function and Atherosclerosis

Abnormal coronary vasomotor function occurs early in the development of atherosclerosis [16] and is typically assessed by studying endothelial-*dependent *epicardial coronary vasodilation by invasive methods [5,17]. However, several x-ray coronary angiography studies [6,10] have also found impairment of endothelial-*independent *coronary vasodilation to NTG in patients, with prognostic significance. In a study of 147 patients with risk factors for CAD (including 9% with diabetes and 84% with angiographic evidence of atherosclerosis) followed for a median 7.7 years, abnormal vasodilator response to acetycholine, cold pressor, and NTG were each independently associated with disease progression and increased cardiovascular events [6]. Consistent with these findings, a study of 163 women (including 26% with diabetes and 45% without angiographic evidence of CAD) found impaired reactivity to NTG and acetycholine in subjects who had future cardiovascular events [10].

### Impaired Coronary Vasomotor Function and Patients with DM and ESRD

Prospective data on coronary vasomotor function are limited in patients with DM [18] and ESRD [19]. However, consistent with an increased cardiovascular risk, *peripheral *vasomotor dysfunction has been almost universally found in these patients [20-26]. Two studies in non-insulin-dependent DM have shown impaired brachial artery vasodilation to both endothelial-dependent and endothelial-independent stimuli [24,26]. Interestingly, in a study using brachial ultrasound in subjects with no documented CAD (including 13.1% with DM), the only risk factor independently associated with impaired NTG-induced vasodilation was DM [27]. Similar to findings in the peripheral vasculature, reduced coronary artery reactivity to NTG has been previously reported in patients with DM. In a study [9] of non-insulin-dependent DM using intravascular ultrasound, coronary artery distensibility and diastolic cross sectional luminal area after NTG were significantly lower in DM compared to controls.

Similar to patients with DM, patients with renal failure have impaired peripheral vasomotor function [19,28,29]. In a study of 28 patients with chronic renal failure (13 on hemodialysis), both flow-mediated and NTG-induced brachial artery vasodilation were impaired to comparable degrees [28]. To date, there are no known studies on coronary vasoreactivity in patients with ESRD.

Our data show that patients with DM and ESRD have impaired NTG-induced coronary vasodilation compared to age-matched controls. The individual data (Figure 3b) reveal that patients generally fell into two groups: those with normal coronary vasodilation (~25%) and those with low coronary vasodilation (< 15%). Eighty percent (80%) of ESRD and 38% of DM patients fell below the 15% threshold, compared to only 6% of the controls.

### Noninvasive Coronary Imaging

CMR [30,31], computed tomography (CT) [32,33], ultrasound [34-36], and nuclear techniques [37,38] all offer alternative approaches to assess coronary artery disease noninvasively. By using sub-mm spatial resolution and analyzing the lumen cross-sectional area, CMR has been shown to have adequate resolution to detect coronary vasodilation to NTG in two prior studies [12,13]. CMR can also directly image the coronary wall, with increased wall thickness demonstrated in patients with Type I DM [39] and non-obstructive CAD [39,40]. CT can provide high-resolution structural imaging of the coronary lumen and wall [32], but the radiation and contrast involved make it suboptimal for serial imaging of coronary vasomotor changes. One recent study did look retrospectively at patients who had more than one coronary CT scan, where NTG was used in one and not in another, and did show significantly larger coronary diameter with NTG [41]. The feasibility of transthoracic echocardiography for measuring epicardial coronary vasodilation has recently been shown in healthy men [42]. The other main approach to assess coronary function noninvasively has been to measure coronary flow or perfusion reserve to a vasodilator stimulus (e.g., adenosine). This is primarily a measure of coronary *microvascular *function in the absence of epicardial stenoses. This can be performed by CMR [30,31,43], positron emission tomography (PET) [37,38], and transthoracic Doppler techniques [34-36] and has been shown to be impaired in patients with coronary risk factors [31,38], including DM[35,38]. More data comparing the prognostic significance of epicardial vs. microvascular vasomotor function are needed.

### Study limitations

A major study limitation is that only endothelium-independent coronary vasodilation with NTG was evaluated. Endothelium-dependent vasomotor function has been shown to be an earlier marker of atherosclerosis and may be more sensitive in patients with subclinical disease [2,4,16]. Future studies should focus on overcoming the challenges of performing a more endothelial-dependent stimulus in the magnetic resonance environment. A preliminary report [44] and a case report [45] on using the cold pressor test show promise [44]. In addition, the size of the clinical cohort was small and recruitment was consecutive, which may have contributed to the significant differences in demographics (i.e., % female) between the two groups. This difference did not account for the finding of impaired vasodilation in the patients, as this finding remained significant even when the only males were analyzed. A study incorporating more female patients is needed to verify if the impaired coronary vasodilation applies to high-risk women. Finally, long-term clinical follow up is needed to determine the prognostic significance of these findings.

## Conclusion

NTG-induced coronary vasodilation assessed noninvasively by CMR was significantly impaired in asymptomatic patients with DM and ESRD. This may provide an additional functional measure of subclinical coronary atherosclerosis in high-risk patients.

## Abbreviations

GE: General Electric; CMR: cardiovascular magnetic resonance; NTG: nitroglycerine; DM: diabetes mellitus; ESRD: end stage renal disease; CSA: cross sectional area; CAD: coronary artery disease; LV: left ventricle; RCA: right coronary artery; LAD: left anterior descending coronary artery; ANOVA: analysis of variance; CT: computed tomography; PET: positron emission tomography

## Authors' contributions

PKN contributed to the study in data collection, data analysis, and manuscript preparation. MT, PY and JE contributed to data collection and data analysis. CHM developed the MR sequences used in the study. MVMC contributed to the study design, data collection, data analysis, and manuscript preparation. All authors have contributed to the manuscript review and editing. All authors have read and approved the final manuscript.
